# Qualitative assessment of take-home naloxone program participant and law enforcement interactions in British Columbia

**DOI:** 10.1186/s12954-016-0106-1

**Published:** 2016-05-21

**Authors:** Andrew Deonarine, Ashraf Amlani, Graham Ambrose, Jane A. Buxton

**Affiliations:** School of Population and Public Health, University of British Columbia, 2206 East Mall, Vancouver, BC V6T 1Z3, Canada; BC Centre for Disease Control, 655 West 12th Avenue, Vancouver, BC V5Z 4R4, Canada

**Keywords:** Naloxone, Take-home naloxone, Law enforcement, Canada, British Columbia

## Abstract

**Background:**

The British Columbia take-home naloxone (BCTHN) program has been in operation since 2012 and has resulted in the successful reversal of over 581 opioid overdoses. The study aims to explore BCTHN program participant perspectives about the program, barriers to participants contacting emergency services (calling “911”) during an overdose, and perspectives of law enforcement officials on naloxone administration by police officers.

**Methods:**

Two focus groups and four individual interviews were conducted with BCTHN program participants; interviews with two law enforcement officials were also conducted. Qualitative analysis of all transcripts was performed.

**Results:**

Positive themes about the BCTHN program from participants included easy to understand training, correcting misperceptions in the community, and positive interactions with emergency services. Potential barriers to contacting emergency services during an overdose include concerns about being arrested for outstanding warrants or for other illegal activities (such as drug possession) and confiscation of kits. Law enforcement officials noted that warrants were complex situational issues, kits would normally not be confiscated, and admitted arrests for drug possession or other activities may not serve the public good in an overdose situation. Law enforcement officials were concerned about legal liability and jurisdictional/authorization issues if naloxone administration privileges were expanded to police.

**Conclusions:**

Program participants and law enforcement officials expressed differing perspectives about warrants, kit confiscation, and arrests. Facilitating communication between BCTHN program participants and other stakeholders may address some of the confusion and remove potential barriers to further improving program outcomes. Naloxone administration by law enforcement would require policies to address jurisdiction/authorization and liability issues.

## Background

Opioid overdose due to illicit drug use and prescription drugs and their associated mortality and morbidity have emerged as a global health issue [1–4]. Globally, an estimated 69,000 people die each year from opioid overdose. However, data from a number of jurisdictions has indicated that community-based naloxone programs, which involve teaching people how to recognize and respond to an opioid overdose, can reduce deaths caused by overdose [5]. In response to these overdoses, community-based take-home naloxone (THN) programs have been developed in a number of different jurisdictions including Canada, the USA, Australia, and several countries in Europe [6–11]. McAuley et al. identified 25 take-home naloxone evaluations and found that every 3 months, there were 5.2–13.1 naloxone administrations for every 100 persons trained [12], indicating the high degree of activity within these programs. In Canada, opioid-related hospital stays have increased by 40 % in the period from 2006 to 2011, making opioids the second-most significant drug category responsible for hospital resource consumption [13], and there have been published evaluations of THN programs in Vancouver [14], Edmonton [15], and Toronto [16]. In addition to THN programs, several jurisdictions have expanded naloxone programs to law enforcement officials, since they are often the first on the scene during an overdose. There are hundreds of police departments now administering naloxone across the USA. According to the North Carolina Harm Reduction Coalition, 654 police departments have naloxone programs across 30 states [17], with the largest number of police departments being located in New York (193), New Jersey (130), and Illinois (58).

The British Columbia take-home naloxone (BCTHN) program was developed by the British Columbia Centre for Disease Control (BCCDC) and initiated in 2012 [18]. At participating community sites, clients are trained to recognize and respond to an overdose and are provided with a naloxone kit (details about the program are available from the program website http://www.towardtheheart.com as of April 14, 2016, there are 178 locations within BC that dispense naloxone as part of the BCTHN program and 581 overdose reversals with naloxone have been reported, 7416 people have been trained, and 7418 people received naloxone kits [19]. Recently, an increase in the number of overdoses associated with fentanyl has been identified in BC [20, 21], creating a higher demand on the program.

Feedback from the BCTHN Community Advisory Board, consisting of THN site coordinators and people who use drugs, have identified some potential concerns related to interactions between program participants and law enforcement. In particular, it was noted from BCTHN records that despite an increased emphasis to call emergency services (calling “911”), nearly a third of those who administered naloxone (herein referred to as “BCTHN naloxone administrators”) did not call 911 [18]. This qualitative evaluation of the BCTHN program was performed to explore participant perspectives on the program and barriers to contacting emergency services during an overdose. Finally, potential barriers to the creation and implementation of programs in which law enforcement officials administer naloxone were explored. By identifying these factors, strategies and policies can be implemented to improve THN programs within BC, throughout Canada and in other jurisdictions.

## Methods

### Ethics, consent, and permissions

Ethics approval was received from the University of British Columbia, Interior Health, and Vancouver Coastal Health ethics boards (H12-02557). All participants provided written informed consent before interviews or focus groups were conducted, and all identifiers were removed from the transcripts.

### Study setting

The BCTHN program is operated by the BCCDC Harm Reduction Program, which develops training materials; is responsible for enrolling sites (i.e., community agencies or health units that partner with health care providers); and distributes naloxone kits to the sites. Enrolled community sites provide training to clients about overdose prevention, recognition, and response and dispense naloxone kits to eligible individuals. Sites report administrative data to BCCDC who also collects details about naloxone administration. BCTHN sites were used to recruit study participants.

### Study design

A qualitative methodology was selected to explore perceptions, practices, and group dynamics that might contribute to the apprehension of calling 911. The study consisted of focus groups conducted with harm reduction clients who had received program training, key informant interviews with experienced BCTHN naloxone administrators, and law enforcement officials. Semi-structured interview guides were adapted from a prior evaluation (by Banjo et al. [14]). In the interview guides, the questions were designed to explore perceptions relating to program training, naloxone administration and overdose response, and attitudes and awareness surrounding the program. These questions were piloted with three people who use opioids in Vancouver. Additionally, questions were created to understand the perceptions that law enforcement officers had about the program, barriers to implementation, community awareness and integration, and compatibility with broader law enforcement policies.

### Participant recruitment

Program participants were recruited from THN sites within the Vancouver Coastal Health, Fraser Health, and Interior Health regions. Focus group participants who used opioids, had received program training, and who were at least 19 years old were eligible to participate in the study. Additionally, client key informants must have administered naloxone at least three times. All clients who participated were provided with a $10 honorarium for their time. Law enforcement participants were recruited by reaching out to existing contacts within two police departments.

### Data collection and analysis

Focus groups and individual interviews with BCTHN naloxone administrators and law enforcement officials took place between April and June 2015. One investigator (GA) conducted individual interviews and focus groups in person and by phone. The interviewer was not otherwise involved in the BCTHN program. Additionally, research assistants were present at the focus groups to assist in the collection of demographic information, to record notes concerning group dynamics and other observable information, and to remunerate participants who left early to avoid disruption of the focus group. Focus groups and interviews took approximately 1 h to complete and were digitally recorded and transcribed verbatim. Paraphrased examples of questions posed in the semi-structured interview include “Did you ever have your naloxone kit confiscated?” or “How did you find out about the training sessions?”

Transcripts were anonymized by removing identifying information and imported into NVivo 10 for analysis. All the authors reviewed the transcripts independently to identify codes for qualitative analysis; the codes were discussed and agreed upon through consensus and themes were developed using a grounded theory approach [22]. Using a content analysis and qualitative descriptive approach (a low-inference analytic method [23]), the themes were then identified in the transcripts. This approach identified information that can be described as “straight and largely unadorned” which can be used for policy development and by practitioners to modify their practice [24]. Other researchers (JAB, AA, GA) reviewed the themes, and differences in opinion were resolved. Member checking with two of the participants was performed to assess the descriptive and interpretive validity of the analysis.

## Results

### Participant demographics

Two focus groups involving eight BCTHN program clients each and four individual interviews with BCTHN naloxone administrators were conducted for a total of 20 client interviewees (Table 1). Two individual interviews were performed with law enforcement officials from BC, one from the Vancouver Police Department and a Royal Canadian Mounted Police officer from Nanaimo.

**Table 1:** Demographics of participating BCTHN program clients (*n* = 20)

Variable	Value
Gender	
Male	11
Female	9
Mean age (years)	
All clients	49
Male	51
Female	46
Education	
Grade 10 or less	8
Grades 11–12	6
Post-secondary	4
Unknown	2
Duration of drug use	
<21 years	4
21–30 years	6
31–40 years	6
>40 years	2
Unknown	2

**Fig. 1:**
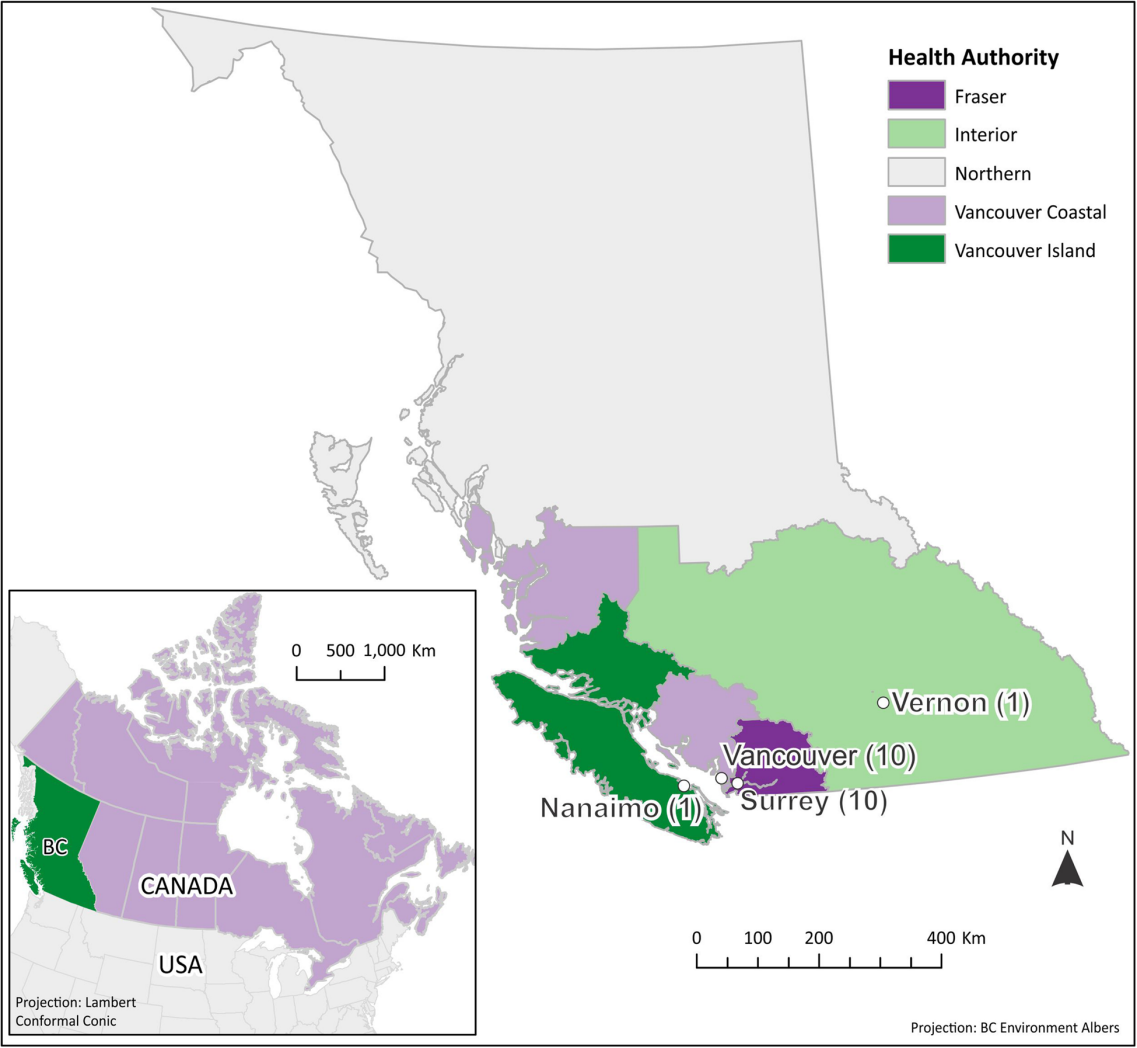
Map of British Columbia showing sites from where the study participants were recruited

The study participants (including BCTHN program participants and police officers) were recruited from various locations in southern British Columbia (see Fig. 1): 10 were from Vancouver and 10 from Surrey (large urban centers) and one from both Vernon and Nanaimo (smaller communities). The interviews in Vernon and Nanaimo were by phone; all other study participants including the focus groups were interviewed in person.

#### General themes from program participants

Program participants expressed positive perspectives about the THN program including the ability to save lives, the understandable nature of the educational content, its accessibility to those with limited reading skills, and integration into the community (see Table 2). Additionally, emergency services were perceived to be very supportive of program participants. One particular benefit that emerged was the correction of false perceptions about naloxone by program participants.

**Table 2:** General themes that emerged from focus groups and interviews with BCTHN naloxone administrators

Theme	Example
Saving lives in the community	“Well we were in an environment where there [were] a lot of lives to be saved so we found it quite beneficial.” - Focus group 1 participant #5
Simple to understand educational content	“Everything was right to the point, what we needed to know and how to use the Narcan kit and how to use, how to open this little bottle without getting cut by your, on your fingers when you open it. So everything was useful in the training.” - Focus group 2 participant #1
Accessibility to people with limited reading skills/illiteracy	“I have problems reading, I am dyslexic. People I know that but, the session itself was really informative and she read it to us and she pointed to it as she was reading it and like explained how to do it, take care of it, physically how, because that’s how I learn things too is by visual.” - Focus group 2 participant #5
Integration into the community	“Round here, most people know who has it usually or they just yell out right, if someone needs it, they just yell out who’s got a kit, right?” - Focus group 1 participant #5
Positive interaction with emergency responders	“I called 911 twice and this was at work, at [agency name]. I naloxoned the one girl and the ambulance came and [said] I did good. And then the second time the police and ambulance came and I did good again, had them all up and ready and they’re like did you do healthcare and I’m like no, I just learned, I’m a self-learner.” - Client interviewee 2
Correcting misperceptions about naloxone	“But people generally are, they accept it with open, with an open mind because – but you know what has misled a lot of people is that movie Pulp Fiction…. Where Buddy gets the adrenalin shot in the heart eh, and people are under the misunderstanding that that was Naloxone or Narcan. So I’ve straightened quite a few people out that have seen that movie.” - Client interviewee 1

#### Arrest warrants and naloxone kit confiscation

BCTHN program participants noted a general concern about being arrested if they called for emergency assistance and law enforcement attended. Police were noted to collect names of those present at the overdose scene and check if they had outstanding arrest warrants. Both law enforcement officials also commented on the issue of warrants, noting that they are aware that it contributes to the apprehension felt by some individuals when interacting with the police (Table 3). Law enforcement interviewees also noted that if they observed large amounts of illegal substances in someone’s home, where an overdose had occurred, they would be obliged to act since possession of illegal substances is a criminal offense. However, they also noted that an overdose is a medical issue and that the public good would be weighed in consultation with commanding officers, making the situation more complex and nuanced.

**Table 3:** THN program participant and law enforcement official perspectives on the themes of warrants, kit confiscation, and arrests

Theme	THN participants	Law enforcement officials
Exercising arrest warrants	“Right away he bolted because he thought 911 had been called and he might have had a warrant and that’s their biggest fear right, they don’t want the police involved. Ambulance, they’re not, they’re not so, it doesn’t matter so much about the ambulance, like they’ll go to the hospital if the ambulance is there. If it’s not there don’t bother, I’m alive now.” - Client interviewee 1	“Typically if we go to an overdose call, it, the fact that somebody’s overdosed doesn’t give us a right in order we just search everybody in the vicinity. I mean really our primary responsibility of this call is to preserve the life of somebody that’s overdosed (right). I, my experience is that the people that are around aren’t necessarily in any peril. I mean we do have to know the last names, etcetera and really unless somebody there happens to have a warrant for their arrest, well I don’t in my experience know that other people that are at the scene are being searched or arrested or detained, or you know unless there’s some reasonable ground that you know that it was you know forcefully administered or you know or anything else they had. I mean that is such a rarity.” - Law enforcement interviewee 2
Arrest for illegal activity (possession, breach of probation, etc.)	“Yeah, police because then like what were you doing when you were coming, what are you doing in this area or were you buying drugs too, so then what if I get in trouble and they start questioning me and them I’m involved for giving her the naloxone, the person the naloxone, and I’m like shit, I was just walking by trying to save a life.” - Client interviewee 2	“But generally, if it’s a medical call, like if, like that’ll, I believe that’ll get fire and ambulance, ambulance for sure, but usually fire tags along for anything. But they’ll go and then I guess if there’s a dangerous circumstance, they’ll call police to assist. What you hear often over police radio is ambulance is attending for an overdose, police [aren’t?] required, just so you’re aware, and then a Sergeant will go, okay, and then that’ll be that, no one’ll go because it’s, it’s kind of fallen now into the realm that even though the drug that was used was illegal, it’s a medical call because where they’re at now is medical…” - Law enforcement interviewee 1
Responder concerns and kit confiscation	“… the first responders showed up and there was, I believe it was an ambulance or a fire, but the guy started yelling at me ‘cause I had the needle in my hand, so I just yelled back at him, for like you know I’m totally, it’s legal for me to have this, why are you mad? Because you can’t carry it? You know so that was probably maybe twice in a situation like that where I‘ve had first responders you know say shit like that to me. Otherwise, nobody’s said anything.” - Client interviewee 4	“I would probably say that if that’s [kit confiscation] happened, it would probably be inexperience of an officer. I mean the Naloxone kits are given by prescription so they can have them, I mean they have no, people can’t get high off them. … I mean we have no business taking those things, that’s those are all measures to preserve their life and health.” - Law enforcement interviewee 2

Another theme that emerged pertained to responder concerns about kit confiscation. One interviewee claimed to have had their kit confiscated while others denied issues with confiscation. However, it is not known if the confiscation occurred early in the program. One law enforcement interviewee suggested that if confiscation occurred, it was probably due to the inexperience of the officer.

#### Law enforcement views on police administering naloxone

Contradictory views of the role of police offers regarding potential naloxone administration were noted (Table 4). One police officer remarked that law enforcement use of naloxone could impinge on the responsibility of other emergency services. However, the other officer noted that ensuring that lives are saved is a shared responsibility of all emergency services, including the police.

**Table 4:** Opposing views on the possible role that police officers can play concerning naloxone administration

“My concern would be that if it’s given to the police, you’re kind of getting into that quasi-territory of cross-training which I know hasn’t always been successful, such as like are you a police officer or you’re a paramedic because generally, [our police officers] rely heavily on their co-relationship with EHS [Emergency Health Services] so if like police officers were to carry kits…” - Law enforcement interviewee 1 “I think the primary mandate, or responsibility of police is to protect public safety and preserve life, and I think that’s a primary role of every emergency responder and I don’t think that police or firefighters or [ambulance] bicker or compete over those roles. We all have that responsibility.” - Law enforcement interviewee 2	

Another theme for law enforcement was that of liability. One law enforcement interviewee noted that liability was an important issue with respect to police administering naloxone in overdose cases (see Table 5).

**Table 5:** Potential concerns about liability discussed by a law enforcement official

“I can see a plethora of issues arising, primarily to do with liability … You know I, the liability issue, in all honesty, that’s I’m sure someone’ll work that out. I’m a, I’m a lowly patrol constable so at that level I could see it being beneficial. … And we’ve recently started carrying tourniquets and you know is there medical concerns if we administer the [inaudible], we’re acting in good faith so you get that kind of pass all.” - Law enforcement interviewee 1	

Additionally, a law enforcement officer articulated concerns about administering naloxone, centering around understanding medical issues and using a needle (see Table 6).

**Table 6:** Concerns about the technicalities of naloxone administration

“The reservations would be that because we’re big city policing, we are, ambulance is always just a moment away, so they say, so for that well then you’re kind of making a decision, am I going to take this medical concern into my own hands, am I properly identifying the issue as an overdose or is the guy having a seizure and this is going to kill him, or I can wait 30 seconds until the ambulance comes, which is fine, which is current practice. I know that generally VPD doesn’t do too much medical, just [inaudible] the primary goal is to get EHS or ambulance there as soon as possible and some very bare bones stuff, so I could see that being complicated. That’s kind of the big decision going in [inaudible] do it now or wait the minute ‘til trained medical personnel can do it. I’ve never given a needle so…” - Law enforcement interviewee 1	

## Discussion

Common themes about naloxone administration were identified from the BCTHN interviewees. The participants provided generally positive perceptions of the BCTHN program, including positive interactions with the first responders. Three factors were suggested to play a role in BCTHN naloxone administrator reluctance to contact emergency services, namely, (1) outstanding warrants; (2) being arrested for illegal activities, such as possession of drugs and breach of conditions of probation; and (3) kit confiscation. We identified no differences in the themes from rural versus urban interviewees.

From the interviews with law enforcement officers, a set of complementary themes was identified. For instance, the police officers noted that while they are required to enforce the law and pursue individuals for outstanding warrants, it can depend on the context. Additionally, the law enforcement officials interviewed noted that in general they would not confiscate naloxone kits in contradiction to the assumptions of some of the BCTHN program participants. The participants’ concern regarding being arrested was contradicted by the information provided by law enforcement: it is often a more nuanced situation in which an officer will consult with a commanding official and weigh the medical and public good of pursuing an arrest in the context of the situation. Communicating to BCTHN naloxone administrators that Vancouver Police Department members do not routinely attend the overdose scenes may address some concerns. Revisiting the general police policy of recording the names of all present at the overdose scene to determine if there are outstanding warrants would help allay fears of arrest. While there are rare instances in which an arrest is required (such as during the case of an assault occurring at the site of an overdose), clear policies outlining when arrests may or may not occur in key scenarios may help law enforcement officials and BCTHN program participants navigate the legal, ethical, and safety issues that can occur during an overdose.

Interestingly, the themes in Table 3 illustrate that the BCTHN program participants and law enforcement officers have differing perspectives on warrants, arrests, and responder concerns with respect to naloxone kit confiscation. One reason for these contradictory opinions may be due to information silos within the BCTHN program participants and other stakeholders such as law enforcement and historic distrust. Hence, clearer lines of communication between program participants and law enforcement could provide significant benefits to the THN program where police could directly dispel false notions and address concerns. Educational efforts within the BCTHN program potentially involving participation by law enforcement community liaisons could help allay fears that program participants have and strengthen the capacity of the program to successfully treat overdoses.

While the law enforcement officials noted that there were major benefits to police administering naloxone, the role of police officers with respect to other first responders emerged as a potential issue of contention. However, the difference in opinion noted in Table 4 could be due to each law enforcement officer being from a different department. A major concern identified by an interviewee was that of liability and the legal consequences an officer might expose themselves to if they were involved in naloxone administration. Concerns around a lack of intranasal delivery (which is used in some jurisdictions in the USA) and about needle delivery were also noted (see Table 6). Clear policies around jurisdiction, interactions with first responders, and also liability would need to be addressed. Davis et al. systematically reviewed the legal issues around liability and authorization with respect to naloxone administration by law enforcement officials. In this study, no officers were sued for administering naloxone, and “Good Samaritan” policies were found to be important in the successful implementation of law enforcement programs [25] which addressed issues of liability and jurisdiction. A recent report on the establishment of a naloxone program for prisoners in New York noted that a key aspect to acceptance of the program among parole officers and corrections staff (who are now being trained for naloxone administration) was understanding the need for naloxone in the community [26]. Establishment of such a program in BC could help achieve the three recommendations made recently by the office of the chief coroner, which consisted of removing barriers to immediate medical assistance after an overdose, raising awareness of the importance of immediate medical attention, and supporting overdose-related interagency learning [27].

Limitations of this study include the potentially limited representativeness of the interviewees, who are predominantly older, long-term drug users (>20 years). Additionally, convenience sampling was used to recruit interviewees which may also make the opinions, themes, and other observations not representative of the program participants as a whole. The researchers involved in the qualitative analysis process attempted to provide an unbiased appraisal of themes and opinions expressed during the interview process. Despite member checking, the role that some of the researchers played in the organizations supporting BCTHN may inadvertently influence results.

## Conclusions

The qualitative assessment of the BCTHN program identified several strengths of the program and potential concerns that may prevent BCTHN naloxone administrators from contacting emergency services during an overdose, such being arrested for outstanding warrants and illegal activity, and respondent concerns about kit confiscation. Instituting clear lines of communication between THN participants and law enforcement with guidelines concerning warrants and kit confiscations could help address issues identified by both parties. With respect to naloxone administration by the police, law enforcement officials identified the issues of liability and jurisdiction/authorization as potential barriers. Based on experience in other jurisdictions especially in the USA, implementing “Good Samaritan” regulations and educational programs emphasizing the benefits of naloxone to the community could be instrumental for such programs going forward in BC, Canada, and other jurisdictions. Discussion and communication about these changes could be facilitated through the use of online portals and other tools.

### Consent to publish

Individual data from participants were not reported, and full permission to use interviewee data (including quotations) as outlined in the manuscript was obtained.

## Abbreviations

BC: British Columbia
BCCDC: British Columbia Centre for Disease Control
BCTHN: British Columbia take-home naloxone
RCMP: Royal Canadian Mounted Police
THN: take-home naloxone
UBC: University of British Columbia

## References

[1] Guidelines for the psychosocially assisted pharmacological treatment of opioid dependence - PubMed - NCBI [http://www.ncbi.nlm.nih.gov/pubmed/23762965]. Accessed 14 Feb 2016. 23762965

[2] Caplehorn JR, Dalton MS, Haldar F, Petrenas AM, Nisbet JG (1996). Methadone maintenance and addicts’ risk of fatal heroin overdose. Subst Use Misuse.

[3] Degenhardt L, Bucello C, Mathers B, Briegleb C, Ali H, Hickman M, McLaren J (2011). Mortality among regular or dependent users of heroin and other opioids: a systematic review and meta-analysis of cohort studies. Addiction.

[4] Dunn KM, Saunders KW, Rutter CM, Banta-Green CJ, Merrill JO, Sullivan MD, Weisner CM, Silverberg MJ, Campbell CI, Psaty BM, Von Korff M (2010). Opioid prescriptions for chronic pain and overdose: a cohort study. Ann Intern Med.

[5] World Health Organization. Community management of opioid overdose. Geneva: WHO; 2014. 25577941

[6] Centers for Disease Control and Prevention (CDC) (2012). Community-based opioid overdose prevention programs providing naloxone—United States, 2010. MMWR Morb Mortal Wkly Rep.

[7] Coffin PO, Sullivan SD (2013). Cost-effectiveness of distributing naloxone to heroin users for lay overdose reversal. Ann Intern Med.

[8] Coffin PO, Sullivan SD (2013). Cost-effectiveness of distributing naloxone to heroin users for lay overdose reversal in Russian cities. J Med Econ.

[9] Tobin KE, Sherman SG, Beilenson P, Welsh C, Latkin CA (2009). Evaluation of the Staying Alive programme: training injection drug users to properly administer naloxone and save lives. Int J Drug Policy.

[10] Maxwell S, Bigg D, Stanczykiewicz K, Carlberg-Racich S (2006). Prescribing naloxone to actively injecting heroin users: a program to reduce heroin overdose deaths. J Addict Dis.

[11] Baca CT, Grant KJ (2005). Take-home naloxone to reduce heroin death. Addiction.

[12] McAuley A, Aucott L, Matheson C (2015). Exploring the life-saving potential of naloxone: a systematic review and descriptive meta-analysis of take home naloxone (THN) programmes for opioid users. Int J Drug Policy.

[13] Young M, Jesseman R. The impact of substance use disorders on hospital use. Ottawa, ON: Canadian Centre on Substance Abuse; 2014.

[14] Banjo O, Tzemis D, Al-Qutub D, Amlani A, Kesselring S, Buxton JA (2014). A quantitative and qualitative evaluation of the British Columbia take home naloxone program. C open.

[15] Dong K, Taylor M, Wild C, Villa-Roel C, Rose M, Salvaggio G, Rowe B (2012). Community-based naloxone: a Canadian pilot program. Can J Addict Med.

[16] Leece P, Hopkins S, Marshall C, Orkin A, Gassanov M, Shahin R (2013). Development and implementation of an opioid overdose prevention and response program in Toronto, Ontario. Can J Public Heal.

[17] US Law Enforcement who carry naloxone » North Carolina Harm Reduction Coalition [http://www.nchrc.org/law-enforcement/us-law-enforcement-who-carry-naloxone/]. Accessed 14 Feb 2016.

[18] Ambrose G, Buxton JA (2015). Overdose recognition and response in the BC take home naloxone program.

[19] Toward the Heart: a Project of the Provincial Harm Reduction Program [http://towardtheheart.com/naloxone/]. Accessed 14 Feb 2016.

[20] Amlani A, McKee G, Khamis N, Raghukumar G, Tsang E, Buxton JA (2015). Why the FUSS (Fentanyl Urine Screen Study)? A cross-sectional survey to characterize an emerging threat to people who use drugs in British Columbia, Canada. Harm Reduct J.

[21] British Columbia Coroners Service. Illicit drug overdose deaths in BC 2006–2015. Burnaby, British Columbia, Canada: British Columbia Coroners Service; 2016.

[22] Martin PY (1986). Grounded theory and organizational research. J Appl Behav Sci.

[23] Sandelowski M (2000). Whatever happened to qualitative description?. Res Nurs Health.

[24] Piper TM, Stancliff S, Rudenstine S, Sherman S, Nandi V, Clear A, Galea S (2008). Evaluation of a naloxone distribution and administration program in New York City. Subst Use Misuse.

[25] Davis CS, Carr D, Southwell JK, Beletsky L (2015). Engaging law enforcement in overdose reversal initiatives: authorization and liability for naloxone administration. Am J Public Health.

[26] Zucker H, Annucci AJ, Stancliff S, Catania H (2015). Overdose prevention for prisoners in New York: a novel program and collaboration. Harm Reduct J.

[27] Egilson M: Preventing death after overdose: BC Coroners Service child death review panel: a review of overdose deaths in youth and young adults. 2009–2013. Vancouver; 2016

